# The Soil Bacterial Communities of South African Fynbos Riparian Ecosystems Invaded by Australian *Acacia* Species

**DOI:** 10.1371/journal.pone.0086560

**Published:** 2014-01-24

**Authors:** Etienne Slabbert, Shayne Martin Jacobs, Karin Jacobs

**Affiliations:** 1 Department of Microbiology, Stellenbosch University, Stellenbosch, Western Cape, South Africa; 2 Department of Conservation Ecology and Entomology, Stellenbosch University, Stellenbosch, Western Cape, South Africa; Institution and Department: Agricultural Research Service, United States of America

## Abstract

Riparian ecosystem along rivers and streams are characterised by lateral and longitudinal ecological gradients and, as a result, harbour unique biodiversity. Riparian ecosystems in the fynbos of the Western Cape, South Africa, are characterised by seasonal dynamics, with summer droughts followed by high flows during winter. The unique hydrology and geomorphology of riparian ecosystems play an important role in shaping these ecosystems. The riparian vegetation in the Western Cape has, however, largely been degraded due to the invasion of non-indigenous plants, in particular *Acacia mearnsii, A. saligna* and *A. dealbata*. This study investigated the effect of hydrology and invasion on the bacterial communities associated with fynbos riparian ecosystems. Bacterial communities were characterised with automated ribosomal intergenic spacer analysis (ARISA) and 454 16S rDNA pyrosequencing. Chemical and physical properties of soil within sites were also determined and correlated with community data. Sectioning across the lateral zones revealed significant differences in community composition, and the specific bacterial taxa influenced. Results also showed that the bacterial community structure could be linked to *Acacia* invasion. The presence of invasive *Acacia* was correlated with specific bacterial phyla. However, high similarity between cleared and pristine sites suggests that the effect of *Acacia* on the soil bacterial community structure may not be permanent. This study demonstrates how soil bacterial communities are influenced by hydrological gradients associated with riparian ecosystems and the impact of *Acacia* invasion on these communities.

## Introduction

Riparian ecosystems are broadly classified as the interface between terrestrial and freshwater aquatic ecosystems [1], [2], [3]. Riparian ecosystems of the South African fynbos play a crucial role in the health and functioning of the diverse fynbos biome as a whole [4]. Fynbos riparian ecosystems have received very little scientific attention compared to the rest of the South African fynbos biome, although they have been shown to contribute disproportionately to ecological processes considering their relatively small land area [5], [6], [7]. These ecosystems are also the site of several important physical and biochemical processes [3], [8]. Riparian ecosystems are unique in the landscape and often exhibit different rates of microbial mediated soil processes compared to upland areas [9]. The most important ecosystem service provided by riparian ecosystems is the supply of clean water, which is greatly, influenced by soil microbial processes [10], [11].

**Figure 1:**
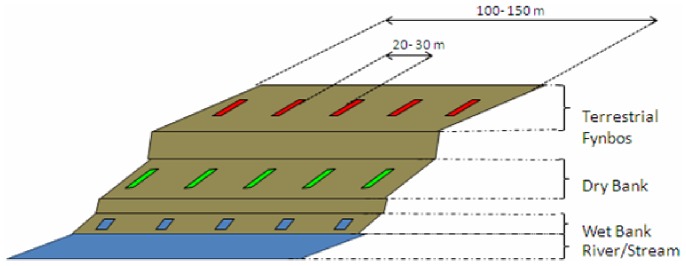
Schematic representation of the lateral zones of the riparian sites and the positions of the sample plots. The extent of each lateral zone is variable depending on the specific dimensions of the riparian zone but ranged from 2m to 10m wide.

Riparian ecosystems associated with the fynbos biome can be easily distinguished from the terrestrial fynbos based on hydrology, geomorphology and the structure of the vegetation [12], [13]. The lateral zones commonly occurring in the fynbos riparian ecosystems are classified as the dry bank and wet bank zones [12] (Figure 1). The dry bank is infrequently inundated, typically only during periods of high flooding, which happen every few years [14]. The water from the river influences the dry bank in the form of ground water during low flow. In the upper catchments (the mountain fynbos riparian ecosystems), high flow and flooding is likely to occur during the rainy winter season [14]. On the other hand, the wet bank zones are classified as the area at the river’s edge, which is under constant influence of the river throughout the year, and is always likely to be moist to wet [12].

The importance of riparian ecosystems and associated plant and microbial biota are now becoming clear due to the increased degradation of riparian ecosystems worldwide [15], [16]. The riparian ecosystems of the Western Cape fynbos biome are largely degraded due to the invasion of non-native *A. mearnsii and A. longifolia* originating from Australia [17] which have, to a large extent, displaced the native riparian vegetation [18]. *Acacia* species such as *A. saligna,* have been shown to be associated with nitrogen-fixing bacteria, which may have a dramatic effect on soil nitrogen stocks and soil microbial community structure [15], [19]. The recovery of the native fynbos vegetation occurs very slowly where Acacias are cleared [20], [21]. This poor recovery sparked interest in the soil dynamics of the fynbos riparian ecosystems, and it has been suggested that soil microbial dynamics may have been modified to the detriment of the restoration of fynbos ecosystem function and structure [22].

Studies have shown that certain bacterial species belonging to the order Rhizobiales may play an important role in the invasiveness of *Acacia* species. [19]. The presence of invasive *Acacias* in South Africa can further be linked to the prevalence of known root associated bacteria, namely *Bradyrhizobium japonicum*, *B. elkanii*, *Rhizobium leguminosarum*, and *R. tropici* that were found to occur in symbiosis with *A. mearnsii* [23]. The effect of invasive *Acacias* on soil microbes has also been observed in other Mediterranean ecosystems [24]. However, most studies, although referring to the importance of soil microbes in the dynamics of invasion by non-native *Acacia*, they have not specifically studied the soil microbial community structure [15].

The main goal of the study was to investigate the effect of non-indigenous invasive *Acacia* spp. on the microbial community structure in riparian zones in the fynbos biome of South Africa. Sharp ecological gradients, laterally across riparian sites, are also included in the study to determine their influence on the soil bacterial community structure.

## Materials and Methods

### Experimental Sites

**Figure 2:**
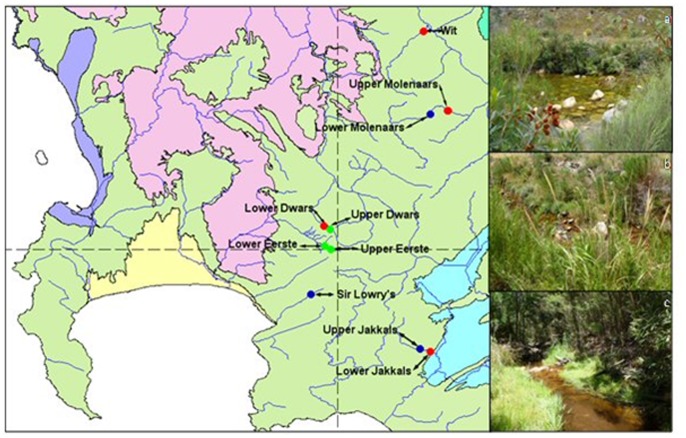
Location of sampling sites with invaded sites indicated in red, cleared sites in blue and pristine sites in green. 2a: Natural riparian vegetation at the lower Eerste River site. 2b: Cleared riparian zone from the Sir Lowry’s and 2c: Riparian zones invaded by Acacia mearnsii at the lower Jakkals River site.

The ten experimental sites are located in the Western Cape, South Africa, and include natural, cleared and invaded riparian ecosystems. A permit was obtained from the conservation authority CapeNature. Permit number: (AAA005-00137-0028). Sampling sites were selected within the upper foothill and adjacent mountain stream areas. Sites with similar vegetation structure and geomorphology according to [18], were selected in order to standardise sampling conditions. The sites included three pristine reference sites flanking the upper Eerste River (UE), lower Eerste River (LE) and the upper Dwars River (UD) (Table 1, Figure 2b). The riparian vegetation of the Molenaars River (UM), Sir Lowry’s River (S) and Jakkals River (UJ) included sites that were clear of non-indigenous vegetation for at least five years (Table 1, Figure 2c). The cleared Jakkals River site experienced a fire event during the winter season and as a consequence those samples were removed from the analysis. The riparian ecosystem adjacent to the Wit River (W) and Jakkals River (LJ) were invaded by *A. mearnsii* and that of the Dwars River (LD) by *A. longifolia* and *A. mearnsii* (Table 1, Figure 2d). The invaded sites were mostly overgrown with woody *Acacias* and the natural fynbos riparian vegetation was almost entirely displaced. Experimental sites adjacent to the rivers were divided into three lateral zones: the riparian wet bank zone, the riparian dry bank zone and the upland terrestrial fynbos zone (Figure 1). The riparian wet bank zone was the area at the edge of the river that was under direct influence of the river water and was characterized by riparian vegetation [13]. This zone is characteristically inundated during all seasons, but dries out to a certain extent during summer. The riparian dry bank zone was also under the influence of the water from the river, although usually by means of groundwater. Typically, the river water only sporadically inundates the dry bank zone during flooding. The terrestrial zone occurs outside the direct influence of the river and excludes plants typical of riparian vegetation [13]. All upland terrestrial sites in the experimental zones consisted of mountain fynbos due to their resistance to invasion and were characterized by Ericoids and Proteas [25].

**Table 1:** Summary of the sample sites indicating the location, position within the catchment, invasive status of the site and the time when the sites were sampled, adapted form Reinecke et

Sample site	GPS		Invaded status	Season 1	Season 2	Season 3
Upper Eerste River (UE)	S 33° 59.803	E 18° 59.056	Natural	√	√	√
Lower Eerste River (LE)	S 33° 59.511	E 18° 58.326	Natural	√	√	√
Lower Dwars River (LD)	S 33° 57′ 14.1″	E 18° 58′ 43,5″	Natural	x	√	√
Upper Jakkals River (UJ)	S 33° 59′ 81.4″	E 18° 59′ 05.3″	Cleared	√	√	√
Upper Molenaars River (UM)	S 33° 44.366′	E 19° 06.805′	Cleared	√	√	√
Sir Lowry′s River (S)	S 34° 05′ 41.5″	E 18° 56′ 39.7″	Cleared	√	√	√
Upper Dwars River (UD)	S 33° 57′ 15.0″	E 18° 58′ 45.1″	Invaded	√	√	√
Wit River (W)	S 33 32′ 18.5″	E 19 10 55.6″	Invaded	√	√	√
Lower Jakkals River (LJ)	S 34° 12.870′	E 19° 11.880′	Invaded	x	√	√
Lower Molenaars River (LM)	S 33° 42.188	E 19° 13.844	Invaded	x	√	x

### Soil Sampling

The first soil samples were collected during March 2010 during the end of the dry autumn season. Sampling plots were positioned to form transects of five, 5 m×5 m plots, located in each lateral zone (Figure 1). Three core samples were taken in the vicinity of the vegetation from each of the five plots using a 25 mm diameter steel cylinder at a depth of 10 cm. This process was repeated for all the plots in the lateral zones and all the experimental sites, resulting in 45 samples per site. The samples were homogenised and DNA extracted within 24h of sampling. The sampling protocol was repeated in August 2010 at the end of the rainy season and again during January 2011 during the dry summer season.

### Abiotic Soil Properties

The abiotic soil properties measured included particle size, available phosphate, pH,, nitrate content, ammonium content, total available nitrogen, total soil nitrogen and soil carbon content. Soil samples were sieved (2 mm) to remove roots and organic debris. The nitrate content, ammonium content and moisture content were determined on fresh soil. The total nitrogen and carbon, and pH were determined on air-dried soil. Bray-2 extractable inorganic P (Pi) was determined on fresh soil with the method described by Bray and Kurtz (1945) [26]. Concentrations of Bray-2 Pi, NO_3_-N, and NH_4_-N were determined colorimetrically with a Genesys 20 spectrophotometer (Thermo Scientific, Waltham, USA). NO_3_-N and NH_4_-N were extracted with 0.5M K_2_SO_4_. For concentrations of NO_3_-N and NH_4_-N, 10 g of soil (<2mm) were placed in 50 ml plastic vials and 25 ml extractant added. The vials were shaken at medium speed for one hour and filtered. NH_4_-N was analysed based on the Berthelot reaction involving phenol [27] and NO_3_-N by nitration of salicylic acid [28]. Total nitrogen and total carbon were analysed by the dry combustion elemental analyser method. Soil pH was measured electrometrically with the Hanna 211 Microprocessor (Hanna Instruments, Woonsocket, USA) in a 1∶2 (w/v, soil: deionised water) slurry [29].

All the multivariate data analysis methods were done using Statistica software v.10 (Statsoft, Tulsa USA). The environmental variables used to compare the sites included available P (ug/g), pH (H_2_0), nitrate concentration (ug/g), total available N (ug/g), total soil nitrogen (%), total soil carbon (%) and the C:N ratio.

### Automated Ribosomal Intergenic Spacer Analysis (ARISA)

DNA was extracted from 0.35 g of fresh soil using the ZR Soil Microbe DNA kit (Zymo Research, California, USA) and the presence of genomic DNA was checked on a 1% agarose gel stained with ethidium bromide PCR reactions were performed using bacterial specific primers for application in ARISA analysis. Bacterial specific primers, ITSReub and FAM (carboxy-fluorescein) labelled ITSF, were used [30]. PCR reactions were done using a GeneAmp PCR System 9700 (AppliedBiosystems, USA). The reaction mixture contained 0.5 µl (±50 ng/ul) of the purified genomic DNA, 500 nM of each primer and 5 µl of 2× KapaTaq Readymix (KapaBiosystems, South Africa) in a total volume of 10 µl. The PCR conditions consisted of an initial denaturing step of 4 min at 95°C followed by 36 cycles of 94°C, for 30 s, 56°C for 45 s and 72°C for 70 s. The reaction was completed with a final extension at 72°C for 5 min and then cooled and held at 4°C. PCR samples were separated on a 1% agarose gel, stained with ethidium bromide and visualized using ultraviolet light. The amplicons from the bacterial specific PCR were run on an ABI 3010×l Genetic analyser to obtain an electropherogram of the different fragment lengths and fluorescent intensities. ARISA samples were run with the ROX 1.1 size standard which varied from 60–1120 bp [31]. ARISA data was analysed using Genemapper 4.1 software (Applied Biosystems), which converted fluorescence data to an electropherogram that represents fragments of different sizes. Only peak height larger than 0.5% of the total fluorescence, ranging from 120 to 1000 base pairs in length, were considered for analysis. A bin size of 3 bp for fragments below 700 bp and 5 bp for fragments above 700 bp was employed to minimise the inaccuracies in the ARISA profiles [32], [31].

### ARISA Data Analysis

The number and relative abundance of OTUs observed with ARISA was used to calculate the Shannon diversity index of all the samples [33]. The Shannon diversity indices were compared using 3 way ANOVA to test for any significant interaction between seasons and the lateral zones and the lateral zones and the invasive status. In cases where significant differences were observed, post-hoc analysis was performed using Tukey’s HDS test for unequal number of samples. ARISA data were used to calculate a Bray-Curtis dissimilarity matrix [34]. The distance matrices were analysed using non-metrical multidimensional scaling (NMDS). The Scree-test was performed to determine the number of dimensions used for every NMDS analysis. The ordination of the NMDS was accepted as informative only when the stress value was below 0.15. The significance and degree of dissimilarity between the Bray-Curtis distances from the samples was analysed based on *a priori* grouping of samples based on the lateral zone and invasive status using ANOSIM [35]. Homogeneity of multivariate dispersion was tested by PERMADISP [36].

The environmental and community data were standardized and log transformed respectively before applying multivariate analysis [37]. Canonical correspondence analysis (CCA) were used to analyze the relationship between abiotic soil variables on the bacterial community structure [38]. The significance of the relationship between soil variables and the community structure obtained from the CCA ordination was tested using 200 permutations [38].

### Pyrosequencing

Due to recognized limitation associated with community fingerprinting methods, rare phylotypes are generally not detected [39]. Consequently, in addition to ARISA, 454 pyrosequencing was used. Based on results from the ARISA analysis, representative samples from invaded, pristine and cleared sites and including all three lateral zones were selected. The samples which were sequenced fell within the 75% confidence limit for the groupings and were randomly selected. Sequencing was done using samples from all three seasons. The hyper-variable regions of V1 to V3 in the bacterial 16S rRNA gene were amplified from extracted community DNA from all samples using the universal bacterial primers 27F and 340R (Table S1) [40]. PCR mixtures of 50 µl volume were prepared using 25 µl KAPA HiFi™ HotStart ReadyMix (Kapa Biosystems, South Africa), 1 µl of each primer (50 *p*mol), 1 µl of the template DNA and 22 µl sterile MilliQ water. PCR reactions were performed using GeneAmp PCR system 9700 (Applied Biosystems, South Africa). The initial PCR was performed under the following conditions: initial denaturation (94°C; 5 min), 25 cycles of denaturation (98°C; 20 sec), annealing (60°C; 15 sec), extension (72°C; 1 min), and a final extension (72°C; 7 min). The PCR products were purified using the DNA clean and concentrator kit (Zymo Research, USA) which served as template for the second round of PCR. The PCR mixture in the second round of PCR was the same as that used for the first reaction. However, during this reaction, the reverse primer 340RA was used, and contained the sequencing adaptor A, an identification key, a unique multiplex identifier (MID) and the universal bacterial primer 340F (Table S1). The forward 27FB primer contained the sequencing adaptor B, an identification key, and the universal bacterial primer 27FB (Table S1). PCR conditions were the same as those used for the first reaction. The PCR amplicons were gel purified using the BioSpin Gel Extraction Kit (BioFlux, Japan). Equimolar concentrations of the PCR amplicons with the different sample-specific barcode sequences (MIDs) were multiplexed and submitted for pyrosequencing (Inqaba Biotec, South Africa) using the 454 GS FLX Titanium Sequencing System (454 Life Sciences, a Roche Company, Branford, USA). The sequence data was submitted to the European Nucleotide Archive (ENA) as PRJEB4470.

### Pyrosequencing Data Analysis

Sequences were filtered, trimmed and assigned to the samples according to their MID sequences. Reads with no undefined base pairs (N’s) and length >50 nt were retained. The MID sequence, key and the reverse and forward primers were trimmed prior to further analysis by using Mothur [41]. Sequences less than 300 nt after trimming were discarded in the analysis. The sequences was screened for chimeras using UCHIME [42]. The sequences were binned into OTUs at 97% sequence similarity, which served as OTUs using cd-hit (Li and Godzik, 2006 [43]). Shannon diversity index and bootstrap and Chao1 richness estimators were calculated with EstimateS [44]. The Shannon diversity indices of the different samples were compared using Kruskal-Wallis ANOVA. A representative sequence for each OTU was selected and aligned using NAST with a minimum alignment length of 150nt and sequence identity of 70% using lanemask PH to screen out hypervariable regions (http://greengenes.lbl.gov/). The clustered OTUs were assigned to taxonomic groups using RDPII taxonomy from the ribosomal database project (RDPII) [45]. RDP training set nine was used, based on nomenclatural taxonomy of Bergey’s Manual, using a minimum confidence threshold of 60%. The alignment was used to construct a distance matrix using the DNAdist function from the PHYLIP 3.6 package [46]. The distance matrix was used to construct a phylogenetic tree using the Jukes–Cantor model. UniFrac analysis was used to overcome the constraints when analysing communities at singular levels of taxonomic classification [47]. The phylogenetic composition of the sample was compared. The Unifrac distances were used to calculate a phylogenetic metric of community similarity. UniFrac distances and measured environmental parameters were correlated using Mantel test. The relative abundances and frequencies of the taxa were used to determine which taxa deliniated the different hydrolohical zones within the riparian ecosytems by performing indicator species analysis with R software using the labdsv package [48]. In addition, indicator analysis was performed using invasive status as the categorical variable.

## Results

### Bacterial OTU Diversity

**Figure 3:**
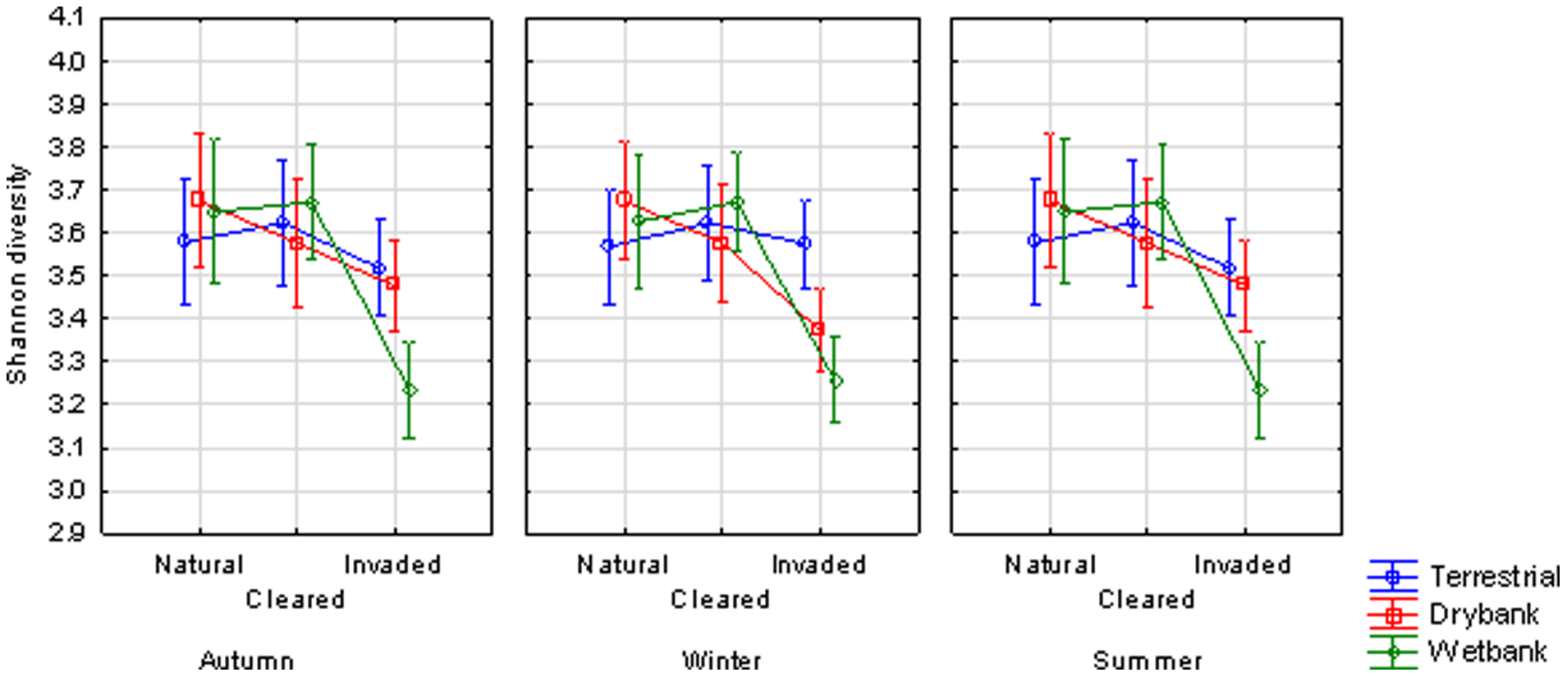
The Shannon’s diversity index based on the bacterial ARISA profiles comparing natural, cleared and invaded lateral zones for all three seasons. The interaction effect between hydrological zones and the invasive status was significant (F = 2.7191, p = .00580).

When comparing the mean Shannon diversity indices within the different lateral zones from autumn, winter and summer samples, no significant differences were observed (p>0.05). The results from the two factorial ANOVA, however, showed that the bacterial diversity of the wet bank zones were significantly lower (p<0.05) when *Acacia* invasion occurred (Figure 3). This decreased diversity was also observed with the pyrosequencing data, which showed a significantly lower Shannon diversity index in the invaded wet bank zones according to Kruskal-Wallis ANOVA (F = 2.54, p = 0.048) (Figure S1).

### Bacterial Community Structure (ARISA)

**Figure 4:**
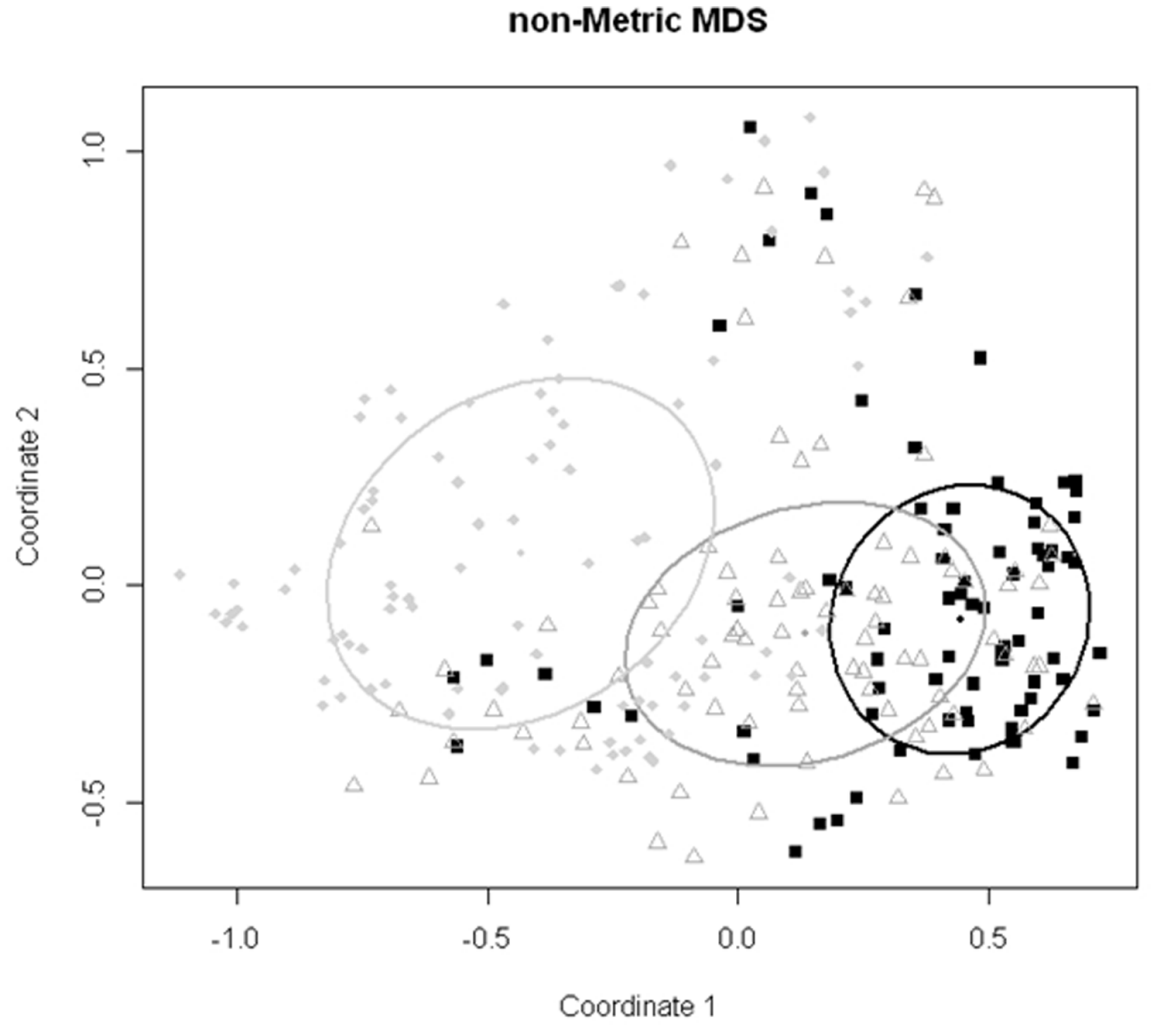
Non-metric multi-dimensional scaling ordination plot of the distance between bacterial communities based on the Bray-Curtis distance. The ellipses represent the samples which were within 75% confidence limit of the centroids and included the wet bank (circles), dry bank (triangles) and the terrestrial samples (squares) (Stress = 0.12).

The NMDS and ANOSIM, comparing all samples, showed significant groupings of the terrestrial, dry bank and wet bank samples over all three seasons (Figure 4, Figure S2). Multivariate analysis, showed significant similarity between samples from the same sites, thus indicating a significant location effect. The effect of lateral zoning was however, the most important factor determining the bacterial community structure (Table 2). PERMADISP dispersions showed that no significant differences in dispersion occurred between sites, however a significant difference in dispersal occurred between the different lateral zones. The ANOSIM post-hoc test showed that the dissimilarity between the terrestrial and wet bank samples were the largest and remained this way over all three seasons (Table 2). Although the dissimilarity between wet bank and dry bank zones was smaller it was still significant (R = 0.28, p<0.05). The dissimilarity between samples from the dry bank and the wet bank increased during the winter season with a increase in the R value from 0.277 to 0.32 (p<0.05) (Table 2). In season three the dissimilarity between the dry bank and wet bank zone again increased to the values seen in season one resulting in a R value of 0.29 between the wet bank and the dry bank zones. The largest dissimilarity still occurred between the terrestrial and the wet bank samples (R = 0.48, p<0.05).

**Table 2:** R values of the ANOSIM comparisons made between hydrological zones and invasion status within dry bank and wet bank zones.

R value	Season 1	Season 2	Season 3
Terrestrial×Wet Bank	0.47	0.48	0.47
Terrestrial×Dry Bank	0.28	0.33	0.27
Dry Bank×Wet Bank	0.32	0.27	0.29
Dry Bank (Natural×Cleared)	0.18*	0.21	0.2
Dry Bank (Natural×Invaded)	0.29	0.22*	0.26
Dry Bank (Cleared×Invaded)	0.26	028	0.2*
Wet Bank (Natural×Cleared)	0.21	0.19*	0.2*
Wet Bank (Natural×Invaded)	0.38	0.4	0.39
Wet Bank (Cleared×Invaded)	0.36	0.37	0.39

Non-significant p values >0.05 are indicated by an asterisk.

**Figure 5:**
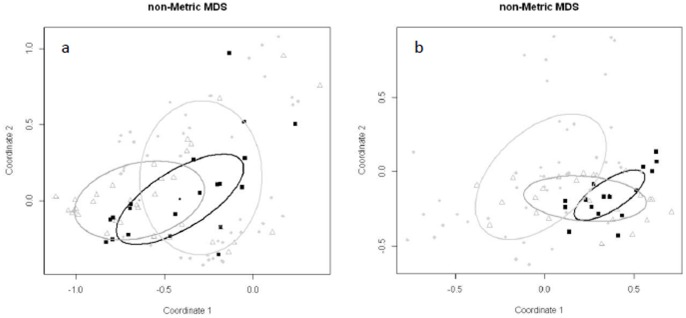
NMDS plot representing the Curtis-Bray distances of the ARISA profiles from the dry bank (a) and wet bank (b) zones. The ellipses represent the samples which were within 75% confidence limit of the centroids and included Pristine (squares), Cleared (triangles) and Invaded (circles) sites (Stress (a) = 0.08, (b) = 0.07).

ANOSIM analysis revealed significant groupings based on the presence of *Acacia* invasion within the dry bank and wet bank zones (Figure 5). The ANOSIM post-hoc test showed that significant differences occurred between the natural and invaded sites and between the cleared and invaded sites (Table 2). The largest difference was seen between the natural wet bank zones and invaded wet bank zones (R = 0.4, p<0.05), followed by the cleared wet bank zone and the invaded wet bank zones (R = 0.39, p<0.05) (Table 2). The differences between the cleared and natural dry bank and wet bank zones were small and only significant for some comparisons (Table 2).

### Pyrosequencing Data

**Figure 6:**
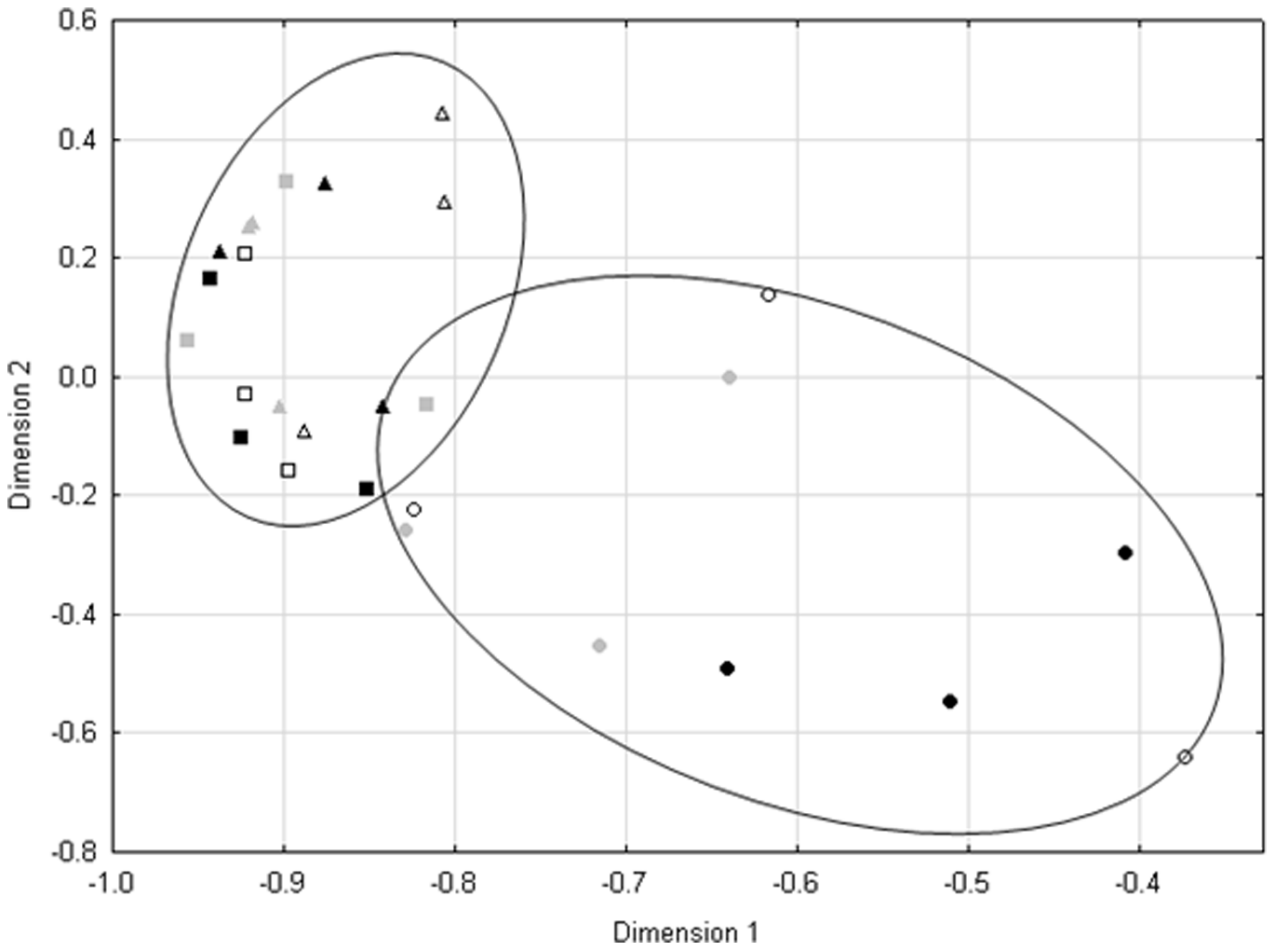
The terrestrial (squares) and dry bank (triangles) form a cluster, which samples showed under dispersion (measured with BETADISP).

The quality filtering of the pyrosequencing reads resulted in 3260 to 12918 quality trimmed sequences per sample, with an average of 5627 reads per sample (Table S2). The average length of the quality filtered pyrosequencing reads was 350 nt. The general composition of the bacterial community on a phylum level was similar, with 18 phyla detected in all the soil samples, six of which were abundant in all the samples. Only eight of the 18 phyla occurred at levels higher than 1% of the total number of reads (Figure S3). The five most abundant phyla accounted for an average of 88.1% of sequence reads. The average representation of phyla in the soil samples included Actinobacteria (32.83%), Alphaproteobacteria (32.2%), Acidobacteria (12.64%), Betaproteobacteria (6.03%) and Planctomycetes (4.39%). When comparing the distribution of phyla between sites (Figure S3), a significantly higher proportion of *Alphaproteobacteria* was observed in the samples from the invaded sites. The proportion of *Actinobacteria* represented in the samples from the invaded sites was, however, lower. The NMDS plot and ANOSIM analysis of the pairwise UniFrac distances showed strong clustering of samples based on the samples’ hydrological location, and not according to invasive status (Table 2, Figure 6).

**Figure 7:**
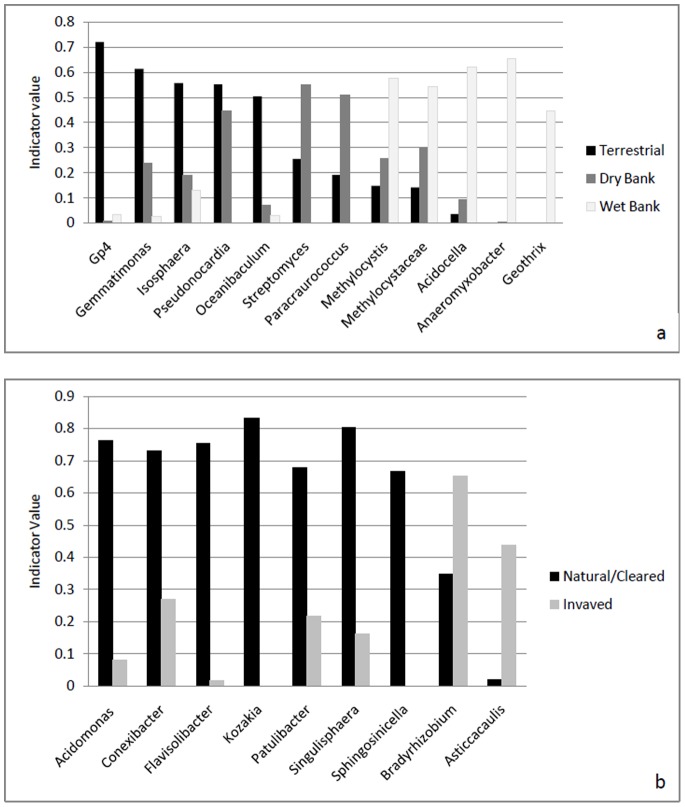
a: The indicator values of the indicator phyla in the community between lateral zones (p<0.05). b: The indicator values and the relative abundance of the indicator phyla in the community when comparing invasive status (p<0.05).

The indicator analysis revealed the significant comparative indicator phyla, values for the different lateral zones (Figure 7a). The genera that were significantly overrepresented in the terrestrial zones were *Gp4*, *Gemmatimonas*, *Isosphaera* and *Oceanibaculum*. The genus that was overrepresented in both terrestrial and dry bank samples was *Pseudonocardia*. The genera that distinguished the dry bank from all other samples were *Streptomyces* and *Paracraurococcus*. The wet bank samples were characterised by the overrepresentation of the genera *Methylocystis*,, *Acidocella*, *Anaeromyxobacter* and *Geothrix*. The phylum *Actinobacteria* was represented to a lesser extent in all the wet bank samples. The genera in this phylum, which were underrepresented in comparison to other lateral zones, include *Conexibacter, Mycobacterium* and *Blastococcus*.

When indicator analysis was performed using the invasion status of the sites, the genus *Bradyrhizobium* occurred at higher frequencies in invaded sites compared to the pristine and cleared sites. The genus *Methylocapsa,* an obligatory methanotrophic gram-negative bacterium [49], also occurred at lower frequencies in the invaded sites (Figure 7b). The phototrophic genus *Rhodopila* [50] showed the same trend. Some groups were present at higher levels in invaded soils compared to pristine and cleared soils. These include the genera *Microvirga* and *Rhizobium,* which are root-nodule forming bacteria [51], *Methylosinus,* and *Methylobacterium,* which are obligatory methanotrophic gram-negative bacteria [52] and *Acidicaldus*, a moderately acidophilic thermophile [53].

### Relationship between Bacterial Community Structure and Abiotic Variables

The PCA analysis showed that total phosphate, nitrate concentration, denitrification rate and total soil carbon explained most of the variation seen in environmental variables between sites (Figure S4, Table S3). The most significant correlations between spesific bacterial genera and environmental variables were with available phosphate, pH and the C: N ratio. These correlations were, however, relatively small (Table S3). The CCA analysis indicated that no significant relationship could be observed between the structure of the bacterial community structure and the soil properties (F = 0.953, p = 0.74) (Figure S5).

## Discussion

### Bacterial OTU Diversity and Richness

The diversity of the bacterial communities (measured as Shannon diversity index) of terrestrial and dry bank lateral zones during all seasons did not differ significantly. The most prominent difference in bacterial diversity was observed in the invaded wet bank zones. In addition to reduced diversity, there was also a reduction in the number of OTUs that could be observed in the invaded sites with the dominance of certain OTUs. This indicates that *A. mearnsii* invasion reduced bacterial diversity, but only under the conditions that occurred in the wet bank zones. The presence of *Acacia* is associated with a decrease in plant diversity which would be expected to cause a reduction in bacterial diversity [54]. The wet bank zones are regularly subjected to flooding and sediment erosion. Studies have shown that such frequent ecological disturbances results in a reduction of bacterial diversity [55].

### Bacterial Community Structure

The environmental gradients across riparian lateral zones, had significant structuring effects on the bacterial communities These included the vegetation structure and hydrology, which involves different frequencies of inundation between the dry bank and wet bank. The wet bank harbour more variable bacterial communities, as showed by the dispersion analysis. The wet bank zone is frequently disturbed by high river flows and previous studies have shown that higher levels of disturbance increases the variability of a community in contras to the more stable communities of the terrestrial samples [56], [57], [58]. The structure of the bacterial community of the dry bank zone during the winter season was more similar to that of the wet bank zone, implying that seasonal changes, is this case higher levels of disturbance by flooding, have an effect on the bacterial community structure. The impact of the ecological gradients associated with riparian lateral zones proved to have a larger effect on the structure of the bacterial communities compared to *Acacia* invasion. Landscape-scale ecological influences have previously been shown to be a determining factor of the microbial community structure [59]. The hydrology of the rivers in this study constitutes a large-scale influence that had a similar structuring effect on the microbial community despite spatial separation and some ecological differences between sampling sites. The ecological influence of the river hydrology is a relatively long term and consistent effect although seasonal. These effects, which shape the different riparian lateral zones, are reflected in the differences in the microbial community structure. The bacterial communities within the three lateral zones remain relatively similar over seasons (Figure S2). This suggests that the long term natural dissimilarity between riparian lateral zones is more important in determining the bacterial community structure than the seasonal differences in this system.

The invasive Acacias affected the structure of the bacterial community of the dry bank and wet bank zones during all the seasons. In invaded sites, the structuring effect of lateral zoning was stronger than the structuring effect of the invasive status (Table 2). This was, however, not observed in the cleared and natural sites, indicating that the invasive *Acacias* are linked with this community shift. During wet conditions, the effect of invasion is visible in the bacterial community structure of the invaded sites. The similarity of cleared and natural sites may be an indication that the community structure of invaded sites shifts back.

### Bacterial Classification

The lower frequency of representatives from the phylum *Actinobacteria* is expected when considering the hydrological properties of the wet bank. The wet banks are generally less well aerated and the phyla *Actinobacteria* and *Acidobacteria* consist of a substantial number of obligatory aerobic bacteria, which was evident in the indicator analysis. Sequence analysis showed that members of the phylum *Alphaproteobacteria* were the dominant group in all the soil samples. *Alphaproteobacteria* was, however, significantly overrepresented in the invaded sites. Although function cannot be inferred on bacterial OTUs when observing bacterial genera, higher frequencies of nitrogen-fixing root associated *Alphaproteobacteria* genera were observed in the invaded wet bank samples [60], [61]. The bacteria most commonly associated with *Acacia* invasion belong to the genus *Bradyrhizobium* [19]. This is consistent with the *Acacia’s* root associated bacteria’s ability to fix nitrogen, which has been suggested to play a role in its invasive ability [15], [62]. The bacterial genera shown to be abundant in the invaded sites also occurred in the natural and cleared sites, although at lower levels.

## Conclusion

Natural fynbos riparian ecosystems are characterised by bacterial communities, which are conspicuously structured by the large-scale hydrology of the area. The structuring effect of the lateral zones is only weakly linked with soil environmental parameters. The experimental design however allowed for the observation the effect of large-scale ecological gradients on the microbial communities structure. Indeed, the general macro-ecological characteristics of the lateral zones across the fynbos riparian ecosystems best explain the bacterial community structure that was observed. Invasion by *Acacia* affected both the diversity and the community structure within invaded wet banks and dry banks. The removal of invasive *Acacia* individuals resulted in the shift of the bacterial communities to their reference or natural state. This is evidence that the bacterial communities within the soil may return to a natural structure if the site remains clear of invasive *Acacias*.

## Supporting Information

Figure S1 **Shannon diversity index of the lateral zones with different invasive status based on pyrosequencing data.** The Shannon diversity of the invaded wet bank zones was significantly lower according to Kruskal-Wallis ANOVA (F = 2.54, p = 0.048).
(TIFF)

Figure S2 **NMDS plot representing the bacterial community structure terrestrial (red), dry bank (green) and wet bank (blue) samples in autumn(triangle), winter (cross) and summer (squares) (Stress = 0.1).**
(TIFF)

Figure S3 **Summary of the distribution frequency of bacterial phyla between natural, cleared and invaded hydrological zones. Only phyla occurring at levels higher than 1% of total reads are shown.**
(TIFF)

Figure S4 **PCA of the soil property data with the bacterial genera plotted as supplementary values (red).**
(TIF)

Figure S5 **The CCA analysis indicated that no significant relationship could be observed between the structure of the bacterial community and the soil properties (F = 0.953, p = 0.74).**
(TIFF)

Table S1 **Pyrosequencing primers used for 454 FLX titanium sequencing.** Primers 340R A1 to A9 contained the universal bacterial 16S rRNA primer 340R, sequencing primer A, an identification key and 9 different multiplex identifiers. Primer 27FB consisted of the universal bacterial 16S rRNA 27F, an identification key and sequencing primer B.
(DOCX)

Table S2 **Table indicating the number of sequences, nonsingleton OTUs, the Chao1 richness estimator, the bootstrap richness estimator and the Shannon diversity index of pyrosequenced samples.**
(DOCX)

Table S3 **Significant Pearson correlations (p<0.05) between enviroenmetal variables and bacterial genera.**
(DOCX)
