# Supplementary figures and images for: The Soil Bacterial Communities of South African Fynbos Riparian Ecosystems Invaded by Australian Acacia Species
Source: PLoS One. 2014 Jan 24;9(1):e86560. doi: 10.1371/journal.pone.0086560 (PMC3901694; doi:10.1371/journal.pone.0086560)

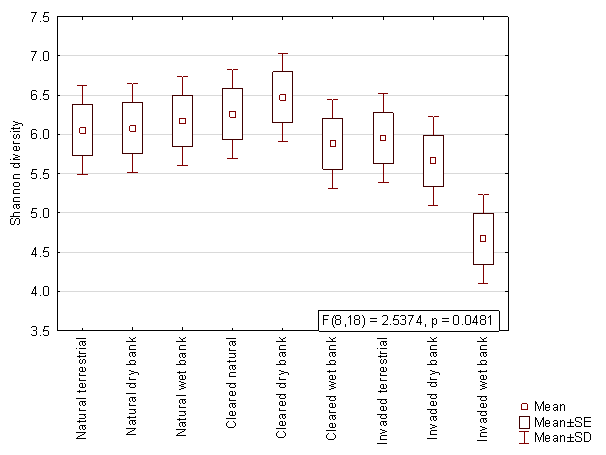

Supplement: Figure S1 — Shannon diversity index of the lateral zones with different invasive status based on pyrosequencing data. The Shannon diversity of the invaded wet bank zones was significantly lower according to Kruskal-Wallis ANOVA (F = 2.54, p = 0.048). (TIFF) [file pone.0086560.s001.tiff]

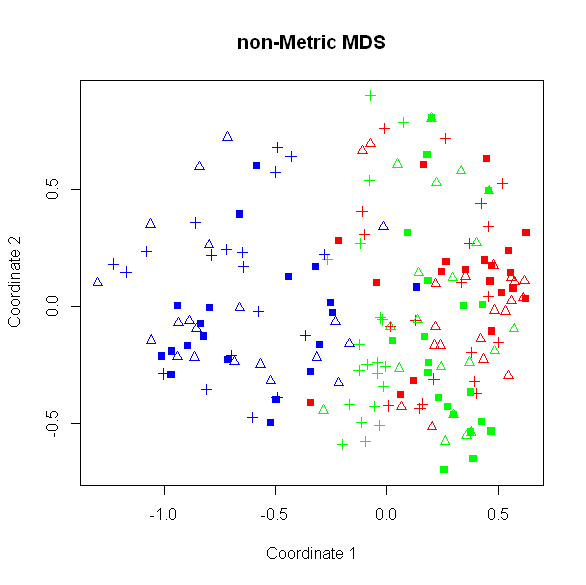

Supplement: Figure S2 — NMDS plot representing the bacterial community structure terrestrial (red), dry bank (green) and wet bank (blue) samples in autumn(triangle), winter (cross) and summer (squares) (Stress = 0.1). (TIFF) [file pone.0086560.s002.tiff]

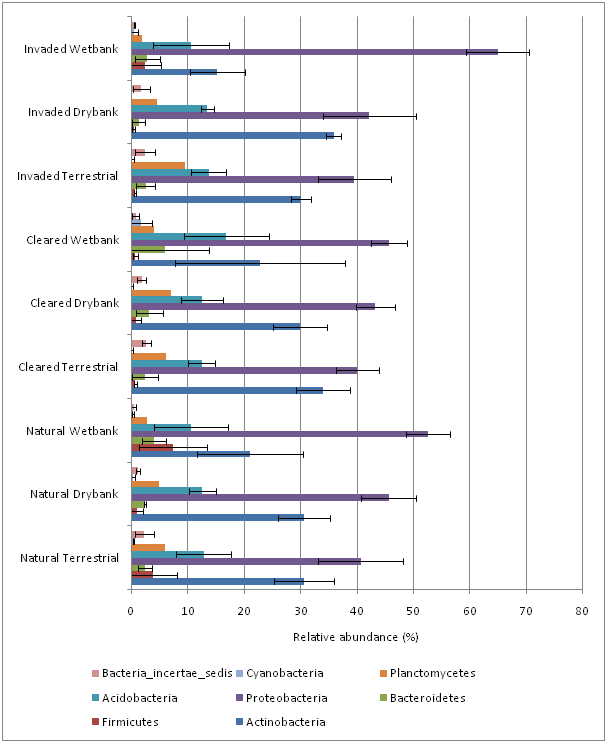

Supplement: Figure S3 — Summary of the distribution frequency of bacterial phyla between natural, cleared and invaded hydrological zones. Only phyla occurring at levels higher than 1% of total reads are shown. (TIFF) [file pone.0086560.s003.tiff]

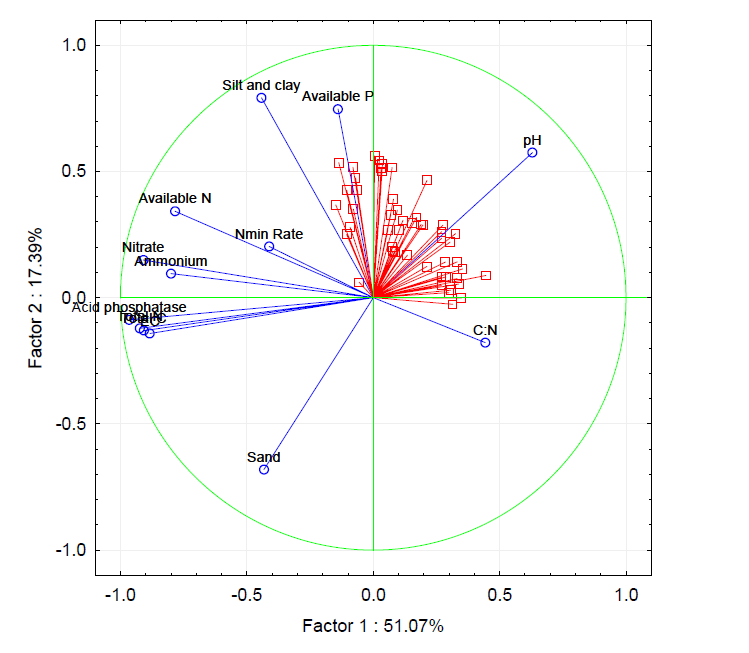

Supplement: Figure S4 — PCA of the soil property data with the bacterial genera plotted as supplementary values (red). (TIF) [file pone.0086560.s004.tif]

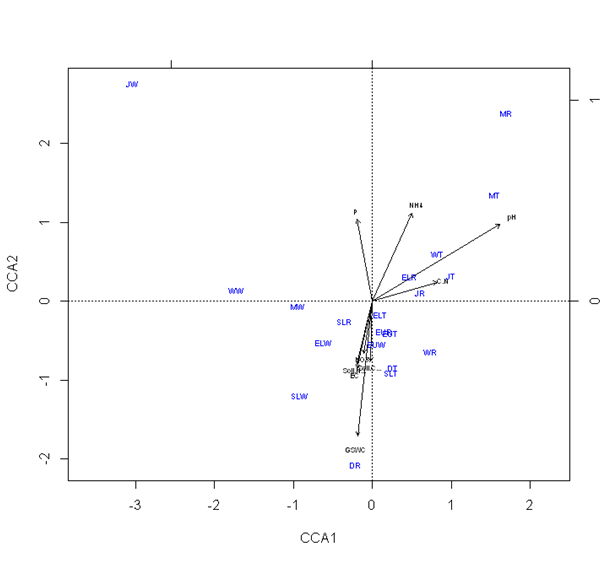

Supplement: Figure S5 — The CCA analysis indicated that no significant relationship could be observed between the structure of the bacterial community and the soil properties (F = 0.953, p = 0.74). (TIFF) [file pone.0086560.s005.tiff]
