# Supplementary material for: The Soil Bacterial Communities of South African Fynbos Riparian Ecosystems Invaded by Australian Acacia Species
Source: PLoS One. 2014 Jan 24;9(1):e86560. doi: 10.1371/journal.pone.0086560 (PMC3901694; doi:10.1371/journal.pone.0086560)
Supplement: Table S1 — Pyrosequencing primers used for 454 FLX titanium sequencing. Primers 340R A1 to A9 contained the universal bacterial 16S rRNA primer 340R, sequencing primer A, an identification key and 9 different multiplex identifiers. Primer 27FB consisted of the universal bacterial 16S rRNA 27F, an identification key and sequencing primer B. (DOCX) [file pone.0086560.s006.docx]

|  | Adaptor A | Key | MID | Specific primer |
| --- | --- | --- | --- | --- |
| 340R_A1 | 5 –CGTATCGCCTCCCTCGCGCCA | TCAG | ACGAGTGCGT | TGCTGCCTCCCGTAGGAGT-3' |
| 340R_A2 | 5 –CGTATCGCCTCCCTCGCGCCA | TCAG | ACGCTCGACA | TGCTGCCTCCCGTAGGAGT-3' |
| 340R_A3 | 5 –CGTATCGCCTCCCTCGCGCCA | TCAG | AGACGCACTC | TGCTGCCTCCCGTAGGAGT-3' |
| 340R_A4 | 5 –CGTATCGCCTCCCTCGCGCCA | TCAG | AGCACTGTAG | TGCTGCCTCCCGTAGGAGT-3' |
| 340R_A5 | 5 –CGTATCGCCTCCCTCGCGCCA | TCAG | ATCAGACACG | TGCTGCCTCCCGTAGGAGT-3' |
| 340R_A6 | 5 –CGTATCGCCTCCCTCGCGCCA | TCAG | ATATCGCGAG | TGCTGCCTCCCGTAGGAGT-3' |
| 340R_A7 | 5 –CGTATCGCCTCCCTCGCGCCA | TCAG | CGTGTCTCTA | TGCTGCCTCCCGTAGGAGT-3' |
| 340R_A8 | 5 –CGTATCGCCTCCCTCGCGCCA | TCAG | CTCGCGTGTC | TGCTGCCTCCCGTAGGAGT-3' |
| 340R_A9 | 5 –CGTATCGCCTCCCTCGCGCCA | TCAG | TCTCTATGCG | TGCTGCCTCCCGTAGGAGT-3' |
|  |  |  |  |  |
|  | Adaptor B | Key |  | Specific primer |
| 27FB | 5' -CTATGCGCCTTGCCAGCCCGC | TCAG |  | AGAGTTTGATCCTGGCTCAG-3' |
