# Supplementary material for: The Soil Bacterial Communities of South African Fynbos Riparian Ecosystems Invaded by Australian Acacia Species
Source: PLoS One. 2014 Jan 24;9(1):e86560. doi: 10.1371/journal.pone.0086560 (PMC3901694; doi:10.1371/journal.pone.0086560)
Supplement: Table S2 — Table indicating the number of sequences, nonsingleton OTUs, the Chao1 richness estimator, the bootstrap richness estimator and the Shannon diversity index of pyrosequenced samples. (DOCX) [file pone.0086560.s007.docx]

| Sample | Season | No. Reads | Number of OTUs | Shannon diversity | Bootstrap | Choa1 |
| --- | --- | --- | --- | --- | --- | --- |
| Eerste River terrestrial | spring | 4072 | 905 | 6.14 | 1469.27 | 1137.57 |
|  | winter | 5680 | 1130 | 6.05 | 913.3 | 1353.41 |
|  | summer | 4348 | 1025 | 5.97 | 865.64 | 1150.36 |
| Eerste River drybank | spring | 12918 | 1110 | 5.25 | 1721.075 | 2184.485 |
|  | winter | 2895 | 895 | 6.96 | 1026.6 | 786.88 |
|  | summer | 6692 | 832 | 6.03 | 874.28 | 1277.01 |
| Eerste River wetbank | spring | 6010 | 1339 | 5.32 | 1491.27 | 1000.56 |
|  | winter | 3795 | 983 | 6.92 | 657.81 | 1328.625 |
|  | summer | 6036 | 1057 | 6.27 | 993.48 | 1962.53 |
| Mollenaars River terrestrial | spring | 4520 | 816 | 6.35 | 1826 | 2548.55 |
|  | winter | 9845 | 946 | 6.61 | 1203.06 | 1781.94 |
|  | summer | 3728 | 816 | 5.82 | 978.63 | 1885.32 |
| Mollenaars River drybank | spring | 9671 | 1348 | 6.15 | 1161.356 | 1497.612 |
|  | winter | 6640 | 1079 | 7.05 | 857.49 | 1555.73 |
|  | summer | 5976 | 1028 | 6.21 | 961.99 | 1591.77 |
| Mollenaars River wetbank | spring | 6403 | 1508 | 5.05 | 1069.93 | 1632.84 |
|  | winter | 2660 | 982 | 6.88 | 1045.38 | 1145.96 |
|  | summer | 3756 | 1042 | 5.71 | 1153.02 | 1259.48 |
| Wit River terrestrial | spring | 3260 | 1078 | 5.79 | 1333.28 | 1105.46 |
|  | winter | 3980 | 1143 | 6.34 | 1146.82 | 1577.34 |
|  | summer | 3652 | 910 | 5.72 | 1252.92 | 1605.1 |
| Wit River drybank | spring | 3348 | 945 | 5.77 | 1250.37 | 1287.846 |
|  | winter | 5910 | 1163 | 5.43 | 801.69 | 1141.79 |
|  | summer | 4036 | 1058 | 5.79 | 984.72 | 894.89 |
| Wit River wetbank | spring | 11647 | 1103 | 4.98 | 1062.015 | 1471.2 |
|  | winter | 4085 | 982 | 4.56 | 907.23 | 730.54 |
|  | summer | 4360 | 1162 | 4.53 | 1371.16 | 1551.77 |

Table S2: Table indicating the number of sequences, nonsingleton OTUs, the Chao1 richness estimator, the bootstrap richness estimator and the Shannon diversity index of pyrosequenced samples.
