# Supplementary material for: The Soil Bacterial Communities of South African Fynbos Riparian Ecosystems Invaded by Australian Acacia Species
Source: PLoS One. 2014 Jan 24;9(1):e86560. doi: 10.1371/journal.pone.0086560 (PMC3901694; doi:10.1371/journal.pone.0086560)
Supplement: Table S3 — Significant Pearson correlations (p<0.05) between enviroenmetal variables and bacterial genera. (DOCX) [file pone.0086560.s008.docx]

Table S3: Significant Pearson correlations (p < 0.05) between enviroenmetal variables and bacterial genera.

| Measured variable | R ^2^ | Genus |
| --- | --- | --- |
| Available P (ug/g) | 0.727 | Iamia |
|  | 0.576 | Patulibacter |
|  | 0.517 | Solirubrobacter |
|  | 0.675 | Streptacidiphilus |
|  | 0.611 | Mycobacterium |
|  | 0.716 | TM7_genera_incertae_sedis |
|  | 0.5 | Mucilaginibacter |
|  | 0.773 | Sorangium |
|  | 0.653 | Burkholderia |
|  | 0.612 | Bradyrhizobium |
|  | 0.622 | Afipia |
|  | 0.554 | Agromonas |
|  | 0.722 | Rhodoblastus |
|  | 0.769 | Methylocystis |
|  | 0.519 | Rhodoplanes |
|  | 0.767 | Caulobacter |
|  | 0.614 | Rhodopila |
|  | 0.707 | Acidisphaera |
|  | 0.707 | Steroidobacter |
|  | 0.638 | Dyella |
|  | 0.619 | Gp3 |
|  | 0.526 | Gp2 |
|  | 0.769 | Gp1 |
|  | 0.691 | Gp14 |
|  | 0.488 | Zavarzinella |
|  | 0.533 | Singulisphaera |
|  |  |  |
| Silt and clay | 0.529 | Streptacidiphilus |
|  | 0.537 | Burkholderia |
|  | 0.508 | Rhodoblastus |
|  | 0.514 | Rhodopila |
|  |  |  |
| pH (H20) | 0.505 | Streptococcus |
|  | 0.493 | Lactobacillus |
|  | 0.524 | Massilia |
|  | 0.566 | Nitrobacter |
|  |  |  |
| C:N ratio | 0.536 | Conexibacter |
|  | 0.506 | Pseudonocardia |
|  | 0.599 | Blastococcus |
|  | 0.498 | Streptococcus |
|  | 0.789 | Nitrobacter |
|  | 0.531 | Agromonas |
|  | 0.714 | Beijerinckia |
|  | 0.755 | Methylovirgula |
|  | 0.633 | Gemmatimonas |
|  | 0.71 | Ktedonobacter |
